# Provider perspectives on constraints in providing maternal, neonatal and child health services in the Lao People’s democratic republic: a qualitative study

**DOI:** 10.1186/1471-2393-13-243

**Published:** 2013-12-27

**Authors:** Vanphanom Sychareun, Sysavanh Phommachanh, Soudavanh Soysouvanh, Chaeun Lee, Minah Kang, Juhwan Oh, Jo Durham

**Affiliations:** 1 University of Health Sciences, Faculty of Postgraduate Study, Vientiane, Laos; 2 Department of Health Policy and Management, Seoul National University, Seoul, Korea; 3 Department of Public Administration, Ewha Womans University, Seoul, Korea; 4 JW LEE Center for Global Medicine, Department of Medicine, Seoul National University College of Medicine, Seoul, Korea; 5 University of Queensland, School of Population Health, Herston, Brisbane, Australia

## Abstract

**Background:**

To reduce its high maternal and neonatal mortality rate and meet Millennium Development Goals four and five, Lao PDR has adopted a national ‘Strategy and Planning Framework of Implementation of Maternal, Neonatal and Child Health Services’. This paper reports on implementation constraints identified in three demonstration sites.

**Methods:**

The objectives of this paper are to analyse health worker perceptions of the implementation of the strategy and constraints faced during implementation. A qualitative design was used with interviews conducted at health facilities in three demonstration provinces. Data were collected through key interviews with provincial/district hospital providers (*n* = 27), health centre staff (*n* = 8) and village health volunteers (*n* = 10). Data was analysed informed by Hanson et al’s health system constraint framework.

**Results:**

In each of the demonstration sites, the Maternal, Neonatal and Child Health program was generally well-understood and the different activities were being implemented. Perceived implementation constraints related mainly to a mix of supply and demand factors. Supply-side constraints related to inadequate human resources, poor remuneration, weak technical guidance, minimal supervision and limited equipment. Demand-side constraints related mainly to cost, limited access to transport, cultural practices and language. Other constraints related to broader strategic management and cross-sectoral contextual constraints. Contextual constraints included low levels of limited education, women’s position in society and poor transport and communications networks. These factors influenced the implementation process and if not addressed, may reduce the effectiveness of the policy and scale-up.

**Conclusion:**

The Lao PDR has a well-defined Maternal, Neonatal and Child Health program. Analysis of the constraints experienced by service providers in implementing the program however, is essential for scaling-up the initiative. To achieve effective implementation and scale-up a number of concurrent interventions are needed to address identified constraints. More research is needed to identify the optimal combination of interventions to improve these constraints. The broader contextual characteristics require longer-term, cross-sectoral action.

## Background

The Lao People’s Democratic Republic (Lao PDR) is a low-middle income country situated in Southeast Asia bordering China, Myanmar, Thailand, Cambodia and Vietnam. Over the last ten years the country has experienced rapid economic growth with an average GDP growth of 8% [1]. On the Human Development Index (HDI) the country is classified as medium, with a HDI of 0.524 ranking it 138 out of 187 countries with comparable data and below the East Asia and the Pacific regional average of 0.671. When adjusted for inequality, the HDI falls to 0.405 [2]. Of the 6.1 million population an estimated 66.8% live in rural areas. Many of these rural areas are difficult to access due to the mountainous terrain and up to 21% of the population lives in areas with no roads [1]. The population is ethnically diverse with 49 officially recognized ethnic groups [1]. Health services are primarily delivered through public sector health centres and district, provincial and central hospitals. A private health sector is emerging but plays a marginal role outside the main urban centres [1].

Poor health status remains a pressing issue. Life expectancy is low at 67.5 years [2]. The Maternal Mortality Ratio (MMR) has declined but remains unacceptably high. The Lao Social Indicator Survey (LSIS) 2011–2012 for example, estimated the MMR to be 357 deaths per 100,000 live births during the seven year period prior to the survey (95% CI 269 – 446) [3]. These estimates suggest the country is unlikely to achieve its fifth Millennium Development Goal (MDG) target of a maternal mortality rate of 260 for every 100,000 live births [4]. The number of child deliveries assisted by health service providers, either at home or at the primary health care or hospital level, was 42 percent in 2012 [3]. Routine Maternal, Neonatal and Child Health (MNCH) reporting suggests that most mothers who die in childbirth do so at home without access to health services [3,4]. The LSIS found the adolescent (15–19 years) birth rate to be 94 births per 1000 live births with substantial differences between rural and urban areas (114 and 44 births per 1,000 adolescents, respectively) [3]. In the two year period preceding the LSIS, 54 percent of women aged 15–49 years who gave birth were reported to have received antenatal care from a health professional. Within this cohort, disparities were evident with women with less education and rural, older and high birth order women. Improvements have been observed however, in the infant mortality rate (IMR) which declined from 123 deaths per 1,000 live births in the mid-1990s to 91 deaths per 1,000 live births in the mid-2000s. This trend has continued with the reported IMR at 68 deaths per 1,000 live births in 2010–11 [3]. If current trends are maintained, the country should achieve its fourth MDG target. The number of children immunized against measles has also improved with 55 percent of children estimated to have been vaccinated by the age of one [3,5].

To accelerate progress in achieving MDG four and five, in 2007 the Ministry of Health (MoH) established a Maternal and Child health - Expanded Program for Immunization Technical Working Group (MCH-EPI TWG) and developed the “Strategy and Planning Framework of Implementation of Maternal, Neonatal and Child Health (MNCH) Services in Lao PDR, 2009-2015” [4]. The MNCH package includes an essential set of demonstrated efficacious interventions for improving MNCH. Services are planned to be person-centred and delivered along the continuum of care from pre-conception, through pregnancy and up to child health care and include a mix of promotive, preventive and curative interventions [4]. In addition, an outreach package of interventions aims to respond to the needs of remote communities [1].

The MNCH strategy has three objectives: 1) improving leadership, governance and management capacity for program implementation; 2) strengthening efficiency and quality of health service provision; and 3) mobilizing individuals, families and communities for maternal, neonatal and child health. The components of objective one include policy review workshops, development of MNCH provincial, district and health centre plans, regular management and technical meetings at the district and health centre level, and improving MNCH information systems. Components of objective two include health staff training, provision of supplies and essential equipment and support for integrated outreach services. Activities related to objective three consist of developing a village health volunteer (VHV)’s manual and a VHV’s kit; community mobilization and introduction of a voucher scheme for the poor to access facility based deliveries.

A demonstration initiative implemented with the assistance of the World Health Organization (WHO) started in December 2009 in two districts. In 2010 it was s scaled up into ten additional districts with a further five districts added in 2011. The initiative is on-going with financial support from Korea Foundation of International Healthcare. A demand-side health financing scheme was a voucher scheme targeting the poor, which entitled the holder to use specific health services without paying the user fee. In some districts, a separate initiative has been the district-based Health Equity Funds (HEF). These funds reimburse selected health care providers for services delivered to eligible poor.

Analysis of health policies and their implementation in low and lower-middle income countries is in its infancy [6]. Yet such analysis is essential to identify factors that can mediate the effectiveness of interventions [6-8]. This paper reports on one component of a larger evaluation of the demonstration program. The evaluation was undertaken to identify constraints and provide recommendations for optimal implementation and scaling-up of the MNCH program. The evaluation is important because despite interventions which reduce maternal and child mortality being known, there is a lack of consensus on an agreed strategy for implementing integrated MNCH care at the primary health care level [9,10]. The specific question the component of the evaluation reported here intended to address ‘What are the constraints identified by the service providers in delivering integrated MNCH services in the demonstration sites?’.

The paper describes the perspectives of the health care providers and makes recommendations for effective scale-up. We drew on Hanson et al’s [7] framework to identify constraints and consider what types of action were needed to effectively scale-up the MNCH program. We defined constraints as the obstacles that restrict or limit the pursuit of desired goals. This includes systems, processes, incentives, and values or norms which can operate at five inter-related levels: the household and community level, the health service delivery level, strategic management, the cross-sectoral level and the broader environment [7]. At the household and community level, constraints relate to demand-side barriers and lack of access to physical, social and financial assets. At the health service delivery level, supply-side constraints relate to quality and distribution of human resources for health, supervision and access to medical supplies [7]. The other three levels of constraints relate in the Hanson et al. framework relate to broader supporting functions which provide an enabling environment and cross-sectoral constraints such as public sector policy, strategic management, communications infrastructure as well as contextual constraints such as political stability, priority given to social sectors and the geographic and physical environment [7,11]. These levels are not mutually exclusive and constraints at one level may affect other levels of the typology. Further as Hanson and colleagues point out, the typology is not definitive and specific constraints may be placed at different levels of the typology [7].

Scaling-up refers to an increase in the level of coverage of a target population [12-14] or increasing the financial, human or capital resources that are required to expand coverage [15,16]. Scaling-up is likely to be more effective if undertaken in a phased manner, beginning with a demonstration program and an evaluation of lessons learned. Typically scaling-up initiatives should take in consideration technical complexity, implementation capacity delivery and usage characteristics [17].

## Methods

### Study design and setting

The project began in 2009 and was expanded in 2010 and 2011. In this study, we evaluated the districts which were started in 2009 and 2010. The sites started in 2011 were excluded due to the short time span of implementation. We used a qualitative approach in exploring health care provider perspectives of constraints in delivering the MNCH program. This approach was deemed to be the most appropriate given this component of the larger study was primarily exploratory [8]. The study setting was the three provinces which have been part of the demonstration that is, Xiengkhouang and Houaphanh in the north and Salavanne in the southeast.

Xiengkouang has a population of 249,817 and more than half of the population belongs to the Hmong ethnic group. Within the province there are eight district health offices/district hospitals and 55 health centres. Each district hospital provides coverage for eighteen sub-districts. The present study was implemented in two of the eight districts, that is, Phoukoud and Khoun districts. Phoukoud district has a population of 28,003 and 63 health staff working at the district health office, district hospital and eight health centres. Of these, 14 work at the district health office, 20 are employed at the district hospital and 29 are employed in the health centres. Khoun district has a population of 33,848 and 68 health staff working in the district. Of these, 21 staff are at the district health office, 21 at the district hospital and 26 are employed in the eight health centres.

Houaphanh province has an estimated population of 280,000. The population is diverse and includes people from the Mien-Yao, Hmong and Tai Daeng ethnic groups. The province has eight district, health offices/district hospitals, eight sub-districts and 54 health centres. The study was implemented at two districts: Xiengkhor and Xamtai. Xiengkhor has one district hospital and seven health centres with 61 health staff serving a population of 25,945. Of these, 28 are employed at the district hospital, 13 are in the district health office and the remaining 20 work in the eight health centres. Xamtai district has a population of 36,540 and is served by 50 health staff (22 at the district hospital, 14 at the health office, 14 at the health centres).

Salavanne province has a population of 336,600. The province has a very diverse multi-ethnic population which includes Kataang, Tai Lao, and the Tai Oy ethnic groups. About 77% of the population lives in rural areas. The study was implemented at Khongsedone district [18]. The district has 53,796 population and 75 health staff (42 staff at the district health hospital, 33 at the health office and 17 at the 8 health centres).

### Study participants

The key informants were health providers at the provincial, district and health center level that were identified by the provincial health departments and district health offices. In each province and in each district, two managers and two technical staff working in MNCH were interviewed. Health providers were from the relevant provinces and districts (Khoun and Kham in Xiengkhouang province; Xiengkhor and Xamtai in Houaphanh; and Khongsedone in Saravanne province). Interviews continued until data saturation had been reached and no new information was forthcoming [8,18]. We approached 47 key informants, two of whom from the provincial health office were unable to participate due to their time availability. In total, 27 Provincial/District hospital providers, eight health centre staff and ten village health volunteers (VHVs) were interviewed.

### Data collection

Qualitative interviews were selected as the data collection method as we aimed to obtain a rich, in-depth description of constraints from the viewpoint of the service providers [8]. The interviews were conducted by a Lao research team from the University of Health Sciences, including the principal investigator (VS) who has extensive experience in qualitative health research in Lao PDR. Interviewers used a semi-structured interview guide. Interviewers were provided a three-day training course prior to data collection in early and mid-January, 2012. The semi-structured interview guide was tested and minor modifications made to ensure that the guide was suitable, culturally acceptable and the words used not too sensitive. Interviews explored participants’ perceptions of the MNCH program (strengths and constraints, major goals of the program and outcomes) and their perspectives of major constraints for mothers’ utilization of MNCH programs and services.

All interviews were audio-recorded with informed consent. The interviewers were not known to the study participants. Participants however, were aware of the interviewers’ status as researchers undertaking an evaluation. Field-notes and memos recorded interview dynamics and reflections on interview accounts [18]. All interviews were transcribed in Lao based on the audio recordings and then translated into English.

### Data analysis

Thematic data analysis, a method for identifying, analyzing and reporting patterns within the data set was used guided by the Hanson et al. [7] framework, particularly the first four levels. VS and SP undertook an initial analysis of the data during the data collection, reviewing the transcripts in Lao reading and re-reading the data, noting down initial ideas based on observation and reading of the transcripts, beginning to identify key themes. Themes were considered significant where there was consistency across and within study participants and/or where they deepened understanding and captured something important in relation to the research question [8]. Subsequently, the researchers reviewed the data collating codes into potential themes, gathering all data relevant to each potential theme and checking if the themes worked in relation to the coded extracts and the entire data set. Throughout the analytic process there was a constant moving back and forward between the entire data set, the coded extracts of data and the on-going analysis of the data [19]. Descriptive codes were used for factual data such as the sex of respondents and their position. The findings contain direct quotes from participants. These quotes have been edited in English for clarity.

### Trustworthiness

As the interviews were conducted and initially transcribed into Lao and then translated into English, there is a potential for misrepresenting intended meaning. To minimize this, translated and edited versions of the quotes were checked with the Lao research team and checked back with the transcripts to check consistency of meaning. The background and methods used have been described to allow readers to assess the applicability. An audit trail has been constructed through memos and field notes. In addition, data collection continued until data saturation was achieved [8,18]. Triangulation of data was achieved by interviewing different people in different locations and use of field notes and memos. Further, preliminary results were presented and discussed in Vientiane, August, 2012 the capital, with stakeholders who work in MNCH.

### Ethics

The Ethics Committee for Health Research, University of Health Sciences, Lao PDR approved the research protocol. Verbal consent was obtained from each participant prior to interviews and the participants were assured of confidentiality. The moderator and interviewer explained the purpose of the study and procedures in Laotian. Participants were also informed that their participation was voluntary and that they could withdraw from the interview/discussion any time without notice in advance and without consequences.

## Results

This section begins by describing the demographic characteristics of the sample. We then outline respondents understanding of the MNCH package and reported improvements since its inception. Then the section discusses constraints to scaling up related to community and household demand-side constraints, service delivery supply-side constraints and management.

### Demographics

In total, 45 health staff was interviewed, of which 13 key informants were provincial health managers and technical staff, 14 district health managers and technical staff, eight were from health centres and ten VHVs. The mean age of the key informants were 38.5 with a range of minimum of 23 years old and maximum 54. Regarding their education and professional training, 31.1% of them were medical doctors, 17.8% were hygiene and sanitation middle level and 15.6% were primary health workers (Table 1).

**Table 1:** Socio-demographic characteristic of the key informants

	**Number**	**Percentage**
**Type of health staff**		
Provincial level	13	28.9
District	14	31.1
Health center	8	17.8
Village Health volunteer	10	22.2
**Age (Mean + SD) (38.5 + 11.3)**		
23–29	8	17.8
30–36	10	22.2
37–43	13	28.9
> = 44	14	31.1
**Sex**		
Male	20	44.4
Female	25	56.8
**Education**		
Medical doctor	14	31.1
Medical assistant	2	4.4
Nurses	3	6.7
Primary health care workers	7	15.6
Hygiene and sanitation middle level	8	17.8
Pharmacy	1	2.2
Primary school	4	8.9
Secondary school	6	13.3

### Understanding of the MNCH program

Most of key informants understood the MNCH package and were able to describe its objectives, activities and training they had received. At the provincial and district levels, main activities were described as follows: 1) improving data collection and management; 2) training in leadership and management for district and health centre staff; 3) training in technical skills; 4) financial support for activities; and 5) mobilizing individuals, families and communities for MNCH. The following quotes help to illustrate provider understanding of the MNCH:

“The goal of the MNCH initiative in Khoun district is to reduce morbidity and mortality among mothers and children, to promote child health from pregnancy through to delivery and improving the health of children under five years old including changing the health behaviour of mothers and increasing the awareness of mothers about health care during pregnancy and nutritional needs of children.” (District Health Officer, Male)

“I have heard of the MNCH programs in my province. The objectives of the MNCH programs are to reduce the maternal mortality. The MNCH activities include: family planning; emergency obstetric care; and training SBA (skilled birth attendants).” (Provincial Health Officer, Male)

Most key informants mentioned common MNCH activities including organizing bimonthly meetings with the health centres and voluntary health workers (VHV), trimonthly meetings with health centre and district health staff, organizing training for skilled birth attendants (SBA) and providing health education on MNCH and MNCH services. Integrated services at the community level were said to include ANC, well-child check up, family planning, child immunization, deworming, and Vitamin A distribution to children.

“The MNCH activities in Houaphanh province consist of promoting people to use the integrated outreach mobile team and providing health education to the villagers; coordinating with the development partners; providing training to the health staff from the provincial, district and health centre level on MNCH; rescheduling of the working system of the integrated outreach mobile services at different levels (provincial, district and health centre levels) and monitoring the MNCH work closely by coordinating with the health staff at different level of Houaphanh province.” (Provincial Health Officer, Female)

The different payment schemes for the poor including use of health equity funds were less understood especially by health workers at the grassroots level as the following quote helps to illustrate:

“I have never heard of the Health Equity Fund. I think it is very good if there is such fund available in our village as it would help our poor people so they could come to health facility for appropriate services. This would help them to have a better life as well as better health.” (Health Center Staff, Female)

Some VHVs had limited understanding of the health insurance schemes. This was mainly because these schemes were not piloted in all districts. There was more understanding about the voucher system by the provincial and district level key informants. The process of learning about the voucher system and how it worked was described by one participant as follows:

*“WHO organized training to provide information about the voucher card system for women in the province. For example, pregnant women attending ANC at least 4 times and having a pink card will receive an incentive for travelling costs based on the distance travelled- up to 3 kms- 10,000 kips, 3–6 kms- 20,000 kips, > 6 kms or higher-50,000 LAK*^*a*^*for the gasoline. In cases of further distance, they will receive no more than 150,000 kips. For a normal delivery, the program will pay the district hospital-175,000 kips and delivery at HC-125,000 kips. The patients also receive 40,000 Kips........In the case of a caesarean the program will pay the hospital-1,500,000 Kips and the food for patients at 40,000 per person. After the WHO training, the provincial authority trained the health staff at the district, HC in order to disseminate to the VHVs to distribute to pink card to the pregnant women in order to promote women to attend ANC and deliver at the health facilities.” (Provincial Health Officer, Female)*

### Improvements in program outcomes

Interpersonal communication and dialogue with clients were reported to have improved. Outcomes reported at the community and household level were mostly positive and included increased health-related awareness and knowledge within the community. There were also reported improvements regarding intervention coverage levels, increased knowledge and skills of health staff in providing MNCH services, participation in MNCH activities among mothers and financial support for mothers. The following quotes illustrate commonly reported outcomes in both demand and supply side:

“People have received more health information and there is an increase in MCH services. The health workers have gained more knowledge about obstetrics and providing health education. They are better and more familiar with the health service utilization and they are able to establish the micro plan without supervision from the provincial level. The health staffs have provided good MNCH services. The VHVs are able to do some basic statistics and collect some data on mothers and children.” (District Health Officer, Male)

“The quality of MCH services has improved and people have more trust in the health staff, the health staff speaks nicely; they have more knowledge and skills and have been trained in SBA modules and other topics of MCH. They are able to provide health education to people.” (Provincial Health Officer, Female)

Some key informants at the district level and health centres felt there were significant improvements at the community level due to the implementation of the MNCH programs.

*“Some improvements that we have observed were: the first prenatal care visit has increased from 73% in 2010 to 87% in 2011. The fourth prenatal care visit has increased from 68% in 2010 to 68.3% in 2011. Delivery at the health centre has increased from 8.6% on 2010 to 21.3% in 2011. Vaccination has almost reached 100% coverage, compared to our goal, vaccination has gradually increased.” (District Health Officer, Male*)

Respondents identified improvements in training, community health education, village level leadership as well as improved skills of the health staff as one person explained:

*“ANC use has doubled. The quality of MNCH services has improved, for example, the training of the SBAs for the HC. They have more knowledge and some skills to provide obstetric gynaecology emergency care which has reduced the number of referrals. For example, before, the HC did not have any delivery and they could not assist with delivery. Right now, they can assist in normal deliveries. Some district hospitals can perform the manual removal of placenta but some still cannot do this. The district health staff can assist with some emergency obstetric care and in case that they cannot assist, they refer to the provincial hospital.” (District Health Officer, Male*)

Other factors external to the health services were also identified as contributing to improved health outcomes. Chief among these were the educational levels of mothers. Several healthcare staff for example, reported that with better access to education, young mothers had higher education than previously and they felt this contributed to a general improvement in health literacy and outcomes. Not all respondents agreed on the extent of improvements however, and some providers especially at the district level, felt that the results were mixed. One of the reasons for these mixed results was out-of-pocket expenses which were reported as being too high for some people.

### Community and household demand-side constraints

Community and household demand-side constraints related to cultural practices, gender roles in decision-making, access to transport and out of pocket expenses.

### Cultural practices

Many respondents reported that demand side constraints related to beliefs that delivering at home was the correct thing to do which was reinforced by community norms and practices. Typically, health care providers stated that it was only when a delivery was not progressing well that help at the clinic or hospital was sought. Some providers reported that the Hmong distrusted caesarean delivery due to their cultural beliefs. In addition, the environment in the health facilities did not match the norm in home-births where ethnic women usually birth alone in the forest. Two different ethnic practices are reported below. While reported as declining, adherence to local beliefs held by the elders, was reported as contributing to the continuation of these practices:

“For ethnic group in Toum Lane, women should deliver their baby in the forest and alone. Women should stay in the forest for 3 days, and only the husband can visit.” (Provincial Health Officer, Female)

“The Hmong ethnic group belief the elders in their clan or in the family/village including cultural beliefs in ghosts/spirits. Sometimes, they perform a spiritual ceremony before coming to the hospital.” (District Health Officer, Female)

In one district, waiting homes had been established for these women. This was reported to have increased their health care utilization rates but this had decreased once the project stopped being externally funded.

### Husband as decision maker

At the household and community level, community participation included health education meetings and home visits by health care providers and village level presentations. Women from the Hmong ethnic group required the permission of their husband and father-in-law for a facility-based delivery. Often this permission was obtained only when it was clear that there were complications, as these informants explained:

“If the husband cannot accompany women, they cannot come as husband drives them or guides them to the hospital.” (Health Center staff, Male)

“If the husband does not agree so his wife also does not come. The husband has a very strong influence on ANC, delivery and family planning.” (District Health Officer, Male)

### Transportation

Lack of transportation, the high cost of available transportation and poor road conditions, especially in the rainy season, was consistently cited as a constraint in women presenting at health care facilities.

“The transportation is still difficult because there are no vehicles to transport patients. For Ta Oi and Samoi districts, the road condition is very poor, especially during the rainy season it is difficult to travel.” (Provincial Health Officer, Female)

Some villages had set up a system whereby a truck or tractor was available to take people to hospital if the patient could pay for the cost for the fuel.

### Out of pocket expenses

The cost of care was a commonly cited constraint. This was not relieved by institutional funds except for the voucher program and equity funds which were available to a few very poor people with certification from the village head. Others depended on social networks for financial support but were generally averse to borrowing money. The most significant costs aside from transportation, were food and medicines. In addition, women needed to have someone to accompany them to health facilities. The following quote helps to exemplify this:

“As I mentioned, money is the main barrier to accessing MNCH services because everything needs to be paid for including travelling, fees of medicines, equipment and other fees, including food and accommodation. If someone is admitted to the hospital, the expenses are not just for the patients, but the expenses are also for relatives to stay with the patients.” (Provincial Health Officer, Male)

### Health service delivery supply-side constraints

Health service delivery supply-side constraints related to shortage of trained staff, language skills of health staff, workloads and access to equipment and medication.

### Shortage of appropriately qualified health care providers

Despite the generally positive comments from the providers that we interviewed, there were remaining concerns for future scale-up of the MNCH programs. A major constraint identified by health service providers was the shortage of appropriate qualified people. Shortages of staff with appropriate training were particularly apparent in the more remote areas and contributed to a situation where many participants reported that positions were held by workers who did not have the appropriate skills for the job to enable them to function efficiently and effectively. The quotes below illustrate commonly reported human resource constraints:

“We need about two more medical doctors. Right now, we have only one medical doctor who is working in obstetric gynaecology, especially we need to have a specialist in obstetric gynaecology. We also need more nurses and midwives - at least four midwives.” (Provincial Health Officer, Male)

In some health centres, it was reported that staff could not assist in deliveries. A related factor was the lack of female healthcare workers and a lack of qualified staff from different ethnic backgrounds who could speak the same language as clients from ethnic groups. In addition, where people were able to access the provincial level hospital, they preferred to do so rather than attend the district level hospital. This was mainly because of the perceived superiority of care at the provincial level. In areas bordering Thailand and Vietnam, it was also reported that women preferred to cross the border for health care either due to perceived quality of care or because they were working there. Casual female labourers in Thailand were also reported as not being able to attend antenatal services in Lao PDR although they often returned home to birth. This also made them unable to access the voucher scheme.

### Language skills of health care providers

As noted above, a lack of language skills was also reported as a common constraint. Many respondents reported challenges in providing an adequate MNCH service and education to people from different ethnic groups. This was particularly the case for female clients who tended to have less Lao language. In such cases, where possible, health care workers asked the women’s husbands to interpret. Typically men were reported as having better Lao language skills as they generally had better education and were more likely to be exposed Lao speakers, for example, in the marketplace.

“The different health seeking behaviour between Hmong and Lao Loum is partly due to Laoloum people can talk easily to the medical doctors as they speak the same language, and are able to read, and write. … Sometimes, we use Hmong women or men who can speak Lao language or sometimes we use the VHVs as translator. Thus, there are some miscommunications and misunderstandings. For example, we told them that pregnant women should not smoke, the Hmong women understood this as pregnant women could smoke.” (Provincial Health Officer, Male)

### Workload of health care providers

A number of respondents reported that the introduction of the MNCH package of program activities had resulted in an excessive number of activities for them to cover. At the community level, health workers reported a lack of a clear and distinct set of tasks compared to prior to the program and reported feeling overburdened by the additional workload. A common sentiment was that the additional activities resulted in them having insufficient time to undertake all the activities effectively. In addition, because undertaking all the activities in villages was time-consuming, in some instances, women could not wait for antenatal checks. As one person explained:

“The workload is too heavy because the provincial health department and the district health office also focus on the health centre including treatment activity, health promotion, and malaria prevention but there are only two health staff at the health center to do this.” (Health Center Staff, Female)

Some respondents stated the increased reporting burden took time away from their direct health care activities. The following respondent reported a common sentiment:

“(The) Data collection form of VHV looks very difficult for me, I do not understand how to fill it in because the form is always changing that makes me confused and I filled in the form wrongly. When I was trained about filling in the form, I understood in one way, but the VHV understood in another way, and when I asked the district health staff, they also understood in another way. We therefore do not know the right way. We had many discussions until a project staff gave us an explanation and made us understood.” (Health Center Staff, Male)

Most respondents recognized the importance of integrated outreach services. Outreach teams were reported as typically composing solely of health centre staff members (which usually means only two individuals) with some assistance from the VHV and community. Most reported difficulties however in performing the integrated outreach due to lack of staff, too many tasks and the skill level of the VHVs who assisted them. Some interviewees felt that two staff members were too few for an outreach team to adequately perform all required services and that at a minimum of three or four were required. These constraints made it hard for the providers to implement all of the MNCH activities as the following typical quotes help to highlight:

“We would like to separate the MNCH integrated outreach activities from the other activities because the workload is too much.” (Health Center staff, Male)

“The VHV do not understand the integrated outreach activities, and the women do not understand the importance of ANC. We have only 2 people doing the integrated outreach activities, however, to do it properly we need to have 5 people.” (Health Center staff, Female)

“The integrated outreach services in our HC are working moderately well as we have small number of staff, and the HC staff and VHV have limited knowledge. If there was training on the integrated activities, it would be good to include taking care of the new born, malpresentation of the child, but I think that the morbidity rate overall has decreased, and the number of pregnant women using the MNCH services at our HC is increased.” (Health Center staff, Male)

“The integrated action is hard work in the first 6 months of the year, since there are a number of tasks that need to be implemented in parallel. We have to collect data with new statistic data and the health volunteers do not understand very well how to fill in the forms.” (Health Center staff, Male)

### Lack of equipment, infrastructure and medicines

The program provided delivery beds*,* weight scales, autoclave and delivery kits for the district hospital and health centres and a computer for the district level health centre. Nevertheless, shortages and a lack of equipment were common themes. Lack of an ambulance was consistently cited, as was a lack of basic health facility infrastructure including places for carers to cook. Not all health centres had refrigerators and where they did, fuel for running them was often only provided during vaccination campaigns. Almost all centres reported they had insufficient delivery kits, scales, stehtoscopes, sphygmomanometers, tongue pressers, and sphygmomanometers for children. Several respondents reported having insufficient vaccines and Vitamin A. One health centre reported they had modified the storage room to be the delivery room and used the office space for antenatal check-ups. The following quotes help to highlight some of the commonly reported problems:

“In general there are difficulties in providing MNCH services due to a lack of some necessary equipment, such as scissors, powder, and other necessary things….” (District Health Officer)

“The infrastructure of the health facilities is underdeveloped. For example, there are no delivery rooms in some health centres and no water supply, although there are some delivery beds. The relatives of patients have to carry the water by themselves.” (Health Center staff, Male)

### Management

Human resource deficiencies at the management level were reported and said to result in inefficient management structures, particularly related to budgeting. At the community level, respondents also reported limited technical guidance and supervision. Respondents frequently reported having to implement activities with insufficient budget and experiencing delays in receiving funding for activities. The following typical quotes help to illustrate this:

“When we received the budget for the outreach activities, we went to do the outreach activities with the district health staff. Sometimes, we received the budget for the outreach integrated services late and we receive the budget after we have submitted the report.” (Health Center Staff, Female)

“The total budget was 2 million kip, but the district office sent to us only 500,000 kip as the first sub-budget, and that was not enough for our work because we had to cover transportation, support for the VHV and the village women who came to help our vaccination activity so we had to use our own money first. After finishing the vaccination activities, we had to submit the expense conclusion to request the outstanding 1.5 million kip.” (Health Center Staff, Male)

Many respondents reported needing more training on administration, filling out forms and budget planning which was seen as complicated with inconsistencies in understanding between VHV and district health staff. The following respondent reported a common sentiment:

“The integrated action is hard work in the first 6 months of the year, since there are a number of tasks that need to be implemented in parallel. We have to collect data with new statistic data and the health volunteers do not understand very well how to fill in the forms.” (Health Center Staff, Male)

Some officers reported problems in coordination and in the partnership between hospitals and provincial MNCH department. This included being given short notice for activities and lack of a plan for activity implementation**.** Some reported communication problems between the provincial health department, district health office and health centres which resulted in delays or postponement of activities without notice.

“The relationship between the MCH provincial department and district MCH division was good but sometimes there are delays of notice and this leads us postponing some activities. So the implementation of the MCH activities was not performed as planned.” (Provincial Health Officer, Male)

“I have seen that there was only one time that the district health staff came to follow up our work on newborn form filling, vaccination, ANC, and to provide some guidebooks about pregnant care for us to read more during the last 3 months.” (Health Center Staff, Male)

## Discussion

The Lao PDR has a well-defined MNCH policy and strategic orientation. These prioritize the development and the implementation of an integrated approach at district hospital level and of a ‘minimum package of health services’ at health centre level. The health service providers in this study generally had a positive perception of the strategy and a good understanding of the main objectives and activities. Some health gains and increased participation were reported through the provision of additional inputs. To maximize outcomes and scale up the intervention, both demand and supply-side barriers need to be sufficiently overcome [20-22]. In addition, the supporting functions, such as strategic management which provide an enabling environment for policy intervention and scaling-up, need to be addressed [7,11].

In this study, constraints were evident at the four levels of the health system included in the analysis (demand and supply-side, strategic management and cross-sectoral). Analysis of the constraints to effective implementation of the MNCH program in the demonstration sites and with implications for scaling-up were consistent with constraints identified in other low and low-middle income countries [7,13,14,17]. At the community and household level, community mobilisation and demand-side constraints resulted in low-uptake of and delayed use of services and poor compliance with treatment. Demand-side constraints included cultural practices and access to physical and financial assets. Out-of-pockets expenses for example were reported to contribute to low access to services. This has been observed elsewhere in Lao PDR particularly with regard to MNCH services [23].

Cultural practices included socio-cultural gender roles. Gender dynamics have also been reported elsewhere as a crucial determinant about decision-making on where women give birth [24,25]. While often the most neglected aspect of primary health care, community participation is essential in addressing demand-side access barriers [26]. Service providers in our study often mentioned that some of the practices of the health facilities, such as having male birth attendants and being examined by male staff made women feel uncomfortable and were frequently in conflict with traditional practices and beliefs. These findings are consistent with other research in Lao PDR [11,22,27,28].

On the supply-side, the commitment of the health staff to provide appropriate services was apparent. Nevertheless, despite having some very competent people, the institutional and human resource capacities for effectively carrying forward MNCH policies were limited. These limitations were observed at the clinical and non-clinical care level. First, there were an inadequate number of staff and second, the number of staff with appropriate training was insufficient. Most of the staff at the different levels had received training but this was often inadequate and with limited follow-up. Further, workloads, especially at the VHV level where VHVs also had to balance their health duties with their livelihood needs, constrained program implementation. Lack of access to necessary equipment and medical supplies inhibited program effectiveness and quality and were often due at least in part, to a poor supply system and financial planning processes. Further, often health staff demonstrated poor understanding of some key policies and standards. These constraints are common in low and lower middle income countries [8,29,30]. Higher level supporting function constraints related to coordination, and management. Shortages in health system managers across the different WHO health system building blocks of finance, information systems and access to appropriate technology and medications are common in low-resource settings [21,29-31]. Indeed, the lack of management capacity may be more critical than the lack of clinical workers and systematic efforts are required to address this constraint [21,31].

Addressing the range of constraints with a suite of interventions is likely to be more effective than targeting single constraints [22]. Some of the constraints to community mobilisation can be addressed by the health sector. Some of the demand–side constraints for example, could be addressed by more consistently taking into account the perspectives of women and communities to ensure health services are culturally and linguistically appropriate. This would help make health facilities an appropriate environment for patients and their carers. In addition, it is important that communities are aware of the availability of financial schemes such as the voucher system and the equity funds and the process to access these are clear and user-friendly. The government’s plans to merge the various financing schemes into one single National Health Insurance Scheme might help address [23]. To be effective however, changes will need to be accompanied by appropriate communication. This might be at least partly addressed by expanding community outreach.

Community outreach is already an important element of the government’s vaccination program, both for campaigns and routine vaccinations. More recently, the MoH has indicated its intention to expand the package of services delivered through outreach, with the aim to increase the coverage of MCH services available at village level, as well as fostering a positive relationship between communities and health service providers [32]. Increased community outreach may also help address delayed decision making by enabling the development of birth and emergency preparedness plans [32]. Other strategies may include improving access to emergency community transport schemes, for example, stretcher teams and transport cooperatives and ensuring appropriate financial support is available for the poor [21].

Our findings suggest scaling-up requires strengthening demand-side constraints through first increasing the size and quality of the workforce. With an average of 2.17 health workers per 1,000 population, Lao PDR health system has a serious shortage of staff and as this study found, in rural areas staff levels are inadequate for effective program implementation [23]. Second, the health workforce needs more pre and in-service training and supervision ideally using standardized training plans [21]. Our findings also suggest that pre and in-service training should include sufficient opportunities to develop and apply midwifery skills during training. Given few women in rural areas deliver in health care centres, this may mean training should be undertaken in urban areas with similar equipment to that available in rural areas. The recently developed National Health Personnel Development Strategy 2009–2020 should begin to address some of these deficits [23].

Given the prevalence of home-birthing, consideration should be given to providing antenatal and postnatal services in communities. This would also mean giving particular attention to strengthening community outreach. Other strategies to improve demand-side constraints include affirmative action to recruit men and women from the relevant ethnic groups [22]. This may necessitate providing additional training in the Lao language prior to pre-service healthcare training. Other strategies to address supply-side constraints include having clear and appropriate administrative, purchasing and supply, budgeting and financial management and clinical guidelines.

Improved management, including supervision and feedback mechanisms can address many of the health system constraints [22]. Given the centrality of the district health offices in effective delivery of the MNCH, at health facilities and community level, strengthening district level management capacity is crucial in scaling-up [23]. Improved salaries and payments would help motivation but need to be linked to broader public sector salary reform. Payment of VHV needs to be considered as they are expected to do more work while at the same time, having to maintain their livelihoods. Clear manuals and guidelines to enable health staff to better manage pregnancy-related complications would also assist [23]. Another important health program input is ensuring that health facilities have the essential equipment and medications to deliver the integrated MNCH package. Addressing these constraints is related to broader national and provincial government budget allocations and government strategies for raising funds for public services. The MoH has to take the lead in advocating for increased budget allocation to health.

Others however will need broader cross-sectoral action. Many of the demand side barriers for example, related to the social determinants of health and are external to the health system [10,33]. Of these, the most important were poverty, road access, education, language and women’s position in society. Similar findings have been reported elsewhere in Lao PDR [27,34]. Other systematic barriers included working as a casual labourer or migrant across the Lao PDR. These constraints cannot be resolved through the investment of additional funds for the health sector, yet are crucial if MNCH service delivery is to be substantially improved [8,21,24]. Demand-side improvements also need to be supported by broader budgeting and policy interventions which allocate funding for training and education for health professionals [21,35]. Improvements in implementation need to be matched by investments in communication and affordable transportation networks. High-level coordination mechanisms already exist and the MoH needs to leverage these mechanisms to advocate for commitments to health producing infrastructure. Table 2 which was informed by the Hanson et al. framework [7], levels one to four and operational solutions within the health care system.

**Table 2:** Key constraints and operational solutions within the health care system

**Level**	**Constraint**	**Cause**	**Solution**
1. Health service delivery level (demand-side)	Shortage of appropriately qualified staff	Inadequate training	Strengthen pre-service and in-service training, develop standardized training plans, use cascade training and increase supervision
			Provide sufficient per diem for supervision
			On-the-job training and mentoring
		Limited number of ethnic group healthcare staff	Human-resource plans including specific affirmative action policies and strategies to attract men and women from different ethnic groups
		Limited number of staff with appropriate language skills	Provide additional education to identified men and women from ethnic groups to facilitate entry into formal training programs
		Poor understanding of some policies and standards	Simple, standardized policies, training and supervision and feedback loops for staff
		Reliance on VHVs	Creation of more paid positions, provide remuneration, per diem for outreach, training and supervision, on-the-job training and mentoring
		VHVs struggle to balance health duties and livelihood needs	
	Insufficient basic supplies, drugs and equipment	Weak supply system an poor financial planning	Strengthen management and supply and provide supervision
			Pre-service and in-service training, supervision and mentoring
			Standardized procurement and disbursement mechanisms
		Distant location of facilities	Deploy trained staff to peripheral health units
			Establish and maintain waiting homes
	Insufficient budget	Insufficient budget allocation	Advocate for increased government budget allocation to health
2. Community and household level (supply side)	Low demand, delayed use of services and poor compliance with treatment	Inadequate affordable transport	Develop community transport schemes
		Household resources and willingness/ability to pay	Develop and promote appropriate finance schemes
		Limited cash flow/livelihood demands – associated with seasonality	Strengthen social health insurance (SHI) and social health protection (SHP) schemes which are inclusive of the poor
		Delayed decision making	Promote birth and emergency preparedness plans
		Language	Strengthen education for ethnic groups
			Recruit from ethnic populations
		Cultural norms	Integrate cultural appropriateness into MNCH program planning and design
3. Policy and strategic management	Weak management, administration and coordination	Few managers	Strengthen district level management capacity
		Limited management training	Clear guidelines and manuals
		High administrative burden relative to skills	Streamline reporting
4. Cross-sectoral,	Limited infrastructure (e.g. electricity, roads, communication networks)	Insufficient cross-sectoral action	Promote cross-sectoral collaboration and strengthen coordination with different technical working groups
	Limited access to education for ethnic groups and women		

### Limitations

This study is one of first to analyze the constraints faced by health care providers in implementing the MNCH strategy in the Lao PDR. As a qualitative study, it is not possible to generalize the results beyond the sites where the data was collected. Nevertheless the data from each of the three districts was remarkably similar and reflects health system constraints identified in other low and low-middle income countries [28]. Further, the objective of this study is not to make statistical inferences but to understand in depth the constraints faced by health care providers in implementing the MNCH strategy and identify areas of intervention to improve effectiveness and optimize potential for successful scale-up.

## Conclusion

To conclude, this study has shown that there are a number of successes, staff generally appreciated the need for the MNCH and there are some workers with a high level of expertise. Nevertheless, there are a myriad of non-mutually exclusive constraints which impede effective delivery and uptake of MNCH. This suggests that a number of concurrent interventions are needed to address demand-side and supply-side constraints as well as building the capacity of the broader supporting health system functions. How these constraints are addressed and prioritized will inevitably be informed in part by political priority, the cultural and geographical context and available resources. On-going monitoring and evaluation is essential to assess the contextual factors which influence the effectiveness and efficiency of interventions designed to address health system constraints. Research is also needed to identify the optimal combination of interventions to improve both demand and supply side constraints. Sustained effort is also needed to address the social determinants of health external to the health system including education, increasing female autonomy and access to social and political capital. Addressing health system constraints including financial constraints may however contribute to addressing some of these barriers.

## Endnotes

^a^1US dollar = 7980 LAK.

## Competing interests

The authors declare that they have no competing interests.

## Authors’ contributions

VS, SP, and CL collected data and did preliminary analysis the data. SP, SS, JO, MK and CL designed the study and guideline for the interview and supervised the data collection. JD, MK, and CL also did preliminary analysis. VS, JD drafted the manuscript. All authors read and approved the final manuscript.

## Pre-publication history

The pre-publication history for this paper can be accessed here:

http://www.biomedcentral.com/1471-2393/13/243/prepub
